# Mechanical Thrombectomy in Patients with Acute Ischemic Stroke and Concomitant Intracranial Hemorrhage

**DOI:** 10.1007/s00062-021-01128-9

**Published:** 2022-01-06

**Authors:** Johannes M. Weller, Julius N. Meissner, Sebastian Stösser, Franziska Dorn, Gabor C. Petzold, Felix J. Bode, GSR-ET investigators, A. Reich, O. Nikoubashman, J. Röther, B. Eckert, M. Braun, G. F. Hamann, E. Siebert, C. H. Nolte, G. Bohner, R. M. Eckert, J. Borggrefe, P. Schellinger, J. Berrouschot, A. Bormann, C. Kraemer, H. Leischner, M. Petersen, F. Stögbauer, T. Boeck-Behrens, S. Wunderlich, A. Ludolph, K. H. Henn, C. Gerloff, J. Fiehler, G. Thomalla, A. Alegiani, J. H. Schäfer, F. Keil, S. Tiedt, L. Kellert, C. Trumm, U. Ernemann, S. Poli, J. Liman, M. Ernst, K. Gröschel, T. Uphaus

**Affiliations:** 1 Section for Vascular Neurology, Department of Neurology, University Hospital Bonn, Bonn, Germany; 2 Department of Neuroradiology, University Hospital Bonn, Bonn, Germany; 3 German Center for Neurodegenerative Diseases (DZNE), Bonn, Germany

**Keywords:** Stroke, Endovascular therapy, Mechanical thrombectomy, Intravenous thrombolysis, Large vessel occlusion

## Abstract

**Purpose:**

Intravenous thrombolysis and mechanical thrombectomy (MT) are standard of care in patients with acute ischemic stroke due to large vessel occlusion. Data on MT in patients with intracranial hemorrhage prior to intervention is limited to anecdotal reports, as these patients were excluded from thrombectomy trials.

**Methods:**

We analyzed patients from an observational multicenter cohort with acute ischemic stroke and endovascular treatment, the German Stroke Registry—Endovascular Treatment trial, with intracranial hemorrhage before MT. Baseline characteristics, procedural parameters and functional outcome at 90 days were analyzed and compared to a propensity score matched cohort.

**Results:**

Out of 6635 patients, we identified 32 patients (0.5%) with acute ischemic stroke due to large vessel occlusion and preinterventional intracranial hemorrhage who underwent MT. Risk factors of intracranial hemorrhage were head trauma, oral anticoagulation and intravenous thrombolysis. Overall mortality was high (50%) but among patients with a premorbid modified Rankin scale (mRS) of 0–2 (*n* = 15), good clinical outcome (mRS 0–2) at 90 days was achieved in 40% of patients. Periprocedural and outcome results did not differ between patients with and without preinterventional intracranial hemorrhage.

**Conclusion:**

Preinterventional intracranial hemorrhage in acute ischemic stroke patients with large vessel occlusion is rare. The use of MT is technically feasible and a substantial number of patients achieve good clinical outcome, indicating that MT should not be withheld in patients with preinterventional intracranial hemorrhage.

**Supplementary Information:**

The online version of this article (10.1007/s00062-021-01128-9) contains supplementary material, which is available to authorized users.

## Introduction

The standard treatment for patients with acute ischemic stroke (AIS) due to intracranial large vessel occlusion (LVO) is mechanical thrombectomy (MT), either alone or in combination with intravenous thrombolysis (IVT) [1, 2]. While the added value of IVT in this setting is currently being investigated (SWIFT-DIRECT, MR CLEAN NO IV, DIRECT MT) [3–5], the benefit of MT has been firmly established by randomized controlled trials [6].

Intracranial hemorrhage encompasses four broad types of hemorrhage: intracerebral hemorrhage (ICH), subarachnoid hemorrhage (SAH), subdural hemorrhage (SDH) and epidural hemorrhage (EDH). These types of hemorrhage differ in etiology, clinical symptoms and prognosis, and outcomes are highly variable. The ICH, SAH and SDH might occur in patients with LVO prior to intervention due to a variety of causes: firstly, LVO patients are frequently on oral anticoagulation due to atrial fibrillation. Oral anticoagulation is a risk factor for cerebral hemorrhage with annual cerebral bleeding rates of 0.23–0.5% for direct oral anticoagulants and 0.7–0.85% for vitamin K antagonists (VKA) [7–10]. Cerebral bleeding, on the other hand, necessitates pausing of oral anticoagulants, which in turn dramatically increase the risk of thromboembolic stroke [11]. Furthermore, intracranial hemorrhage and AIS share nonmodifiable risk factors such as age, sex, ethnicity or previous stroke, and modifiable risk factors including arterial hypertension, hyperlipidemia, smoking, obesity and alcohol consumption [12]. Secondly, in acute LVO, sudden-onset paresis or unconsciousness might cause the patient to fall and lead to traumatic intracranial hemorrhage. Thirdly, intracranial hemorrhage is also a complication of IVT, affecting 8.8% of stroke patients in the ECASS II trial [13]. In patients treated with the drip-and-ship paradigm, IVT-associated intracranial hemorrhage can be detected before MT [14, 15].

With increasing MT numbers over the last years reaching, e.g. 16,135 cases (7.2% of hospitalized AIS patients) in Germany in 2019 alone, one would expect to see patients with LVO and preinterventional intracranial hemorrhage now and then, at least in larger stroke centers [16]. While IVT is obviously contraindicated in this setting, the optimal treatment of these patients currently remains unknown as randomized controlled MT trials excluded patients with intracranial hemorrhage [17–21]. Preliminary insight into MT in patients with preinterventional intracranial hemorrhage is based on published anecdotal data, including six patients with ICH after IVT [14, 15], two patients with spontaneous ICH [22, 23], three patients with SDH [24] and two with SAH [15, 25]. Therefore, the goal of this study was to describe a larger cohort of patients undergoing MT with AIS due to LVO and preinterventional intracranial hemorrhage.

## Methods

The German Stroke Registry—Endovascular Treatment (GSR-ET; NCT03356392) is an ongoing, prospective, open-label, multicenter registry of patients with AIS due to LVO treated by MT. Patients were prospectively recruited between July 2015 and December 2019 from 25 German hospitals. A detailed description of the trial has been published previously [26]. Medical information was reviewed of all patients with intracranial hemorrhage as reported by the database or if given as a reason for omitting or stopping IVT. Patients were retrospectively included in this analysis if intracranial hemorrhage was detected on neuroimaging before initiation of MT. Patient characteristics were extracted from the registry. Functional independence was defined as a modified Rankin scale (mRS) score of 0–2. A 4:1 propensity score matching (R version 4.0.3, R core team 2020, package MatchIt, R Foundation for Statistical Computing, Vienna, Austria. https://www.R-project.org/) was performed to select a cohort of patients without intracranial hemorrhage matched for age, sex, premorbid mRS and NIHSS with the nearest-neighbour approach. All other analyses were performed with SPSS (version 25, IBM Corp, Armonk, NY, USA). Standard descriptive statistics were provided and parameters were compared between groups by Student’s t‑test, Mann-Whitney U test, and Fisher’s exact test, where appropriate. Significance level was set to α < 0.05 and all tests were two-sided.

## Ethics Approval

Data collection was approved by the Ethics Committee of the University Munich, Germany (689-15) and Ethics Committees at the participating centers according to local regulations.

## Results

Among 6635 patients in the registry treated with MT, 32 AIS patients with LVO and preinterventional intracranial hemorrhage were identified, resulting in a prevalence of 0.5%. The median age was 79.5 years (interquartile range [IQR] 75–83.75 years) and 50% were female (Table 1). The rate of pretreatment functional independence (mRS ≤ 2) was 56.3%.

**Table 1:** Baseline, periprocedural and outcome characteristics of all patients

	Age (years)	Sex	Hemorrhage	Side of hemorrhage	Bleeding cause/risk factor	OAC	Interval days	Premorbid mRS	NIHSS	ASPECTS	Occluded vessel	Ipsilateral	IVT	Final mTICI	Stroke etiology	Discharge mRS	d90 mRS
1	80	f	ICH	Right	Trauma	VKA	5	0	27	NA	BA/VA	NA	No	2b	CE	6	6
2	90	f	ICH	Left	OAK	VKA	9	5	15	10	Left M2	Yes	No	0	CE	6	6
3	72	m	ICH	Right	OAK	NOAC	7	0	17	NA	Right M1	Yes	No	3	CE	5	5
4	75	m	ICH	Left	IVT	No	0 ^a^	0	7	8	Right M1	No	Yes	3	CE	0	0
5	86	m	ICH	Left	IVT	No	0 ^a^	4	20	9	Left M1	Yes	Yes	2b	CE	5	6
6	76	f	ICH	Left	IVT	No	0 ^a^	0	13	7	Right M1	No	Yes	2b	CE	0	0
7	89	f	ICH	Right	Ischemia	No	0 ^a^	0	29	NA	BA	NA	No	3	CE	3	2
8	82	m	ICH	Right	Ischemia	No	7	2	22	10	Left M1	Yes	No	3	CE	5	6
9	77	m	ICH	Bilateral	Cavernoma	No	7	1	8	10	Left M2	Yes	No	2b	CE	2	Uk
10	76	m	ICH/SAH	Bilateral	Trauma	No	0 ^a^	0	10	8	Left M1	Yes	Yes	2b	CE	2	1
11	83	f	ICH/SAH	Right	OAK	NOAC	Uk ^b^	3	16	8	Left M1	No	No	2b	CE	5	6
12	80	f	ICH/SAH	Right	IVT	No	0 ^a^	0	7	10	Left M2	No	Yes	2b	LAAS	5	Uk
13	92	f	SAH	Right	Trauma	No	17	3	19	Uk	Left M2	No	No	2b	CE	4	4
14	73	m	SAH	Right	IVT	No	0 ^a^	0	5	10	Right M2	Yes	Yes	2b	CE	1	0
15	77	m	SAH	Bilateral	Aneurysm	No	0 ^b^	0	32	NA	BA	NA	No	2b	SUE	6	6
16	82	f	SAH	Left	OAK	NOAC	0 ^b^	3	15	10	Left M1	Yes	No	0	CE	6	6
17	73	m	SAH	Left	Trauma	No	10	2	15	8	Left ACI/M1	Yes	No	0	LAAS	5	6
18	63	f	SAH	Left	Trauma	No	0 ^a^	0	10	8	Left ACI	Yes	No	2b	LAAS	2	2
19	93	m	SAH	Right	IVT	No	0 ^a^	0	7	10	Right M1	Yes	Yes	3	CE	4	5
20	79	f	SAH	Left	Trauma	No	0 ^a^	4	28	NA	BA	NA	No	3	CE	5	6
21	82	f	SAH	Left	Trauma	No	1	0	15	8	Left M1	Yes	No	3	CE	4	4
22	86	f	SAH	Right	Trauma	No	0 ^a^	1	10	10	Left M1	No	No	3	UE	4	6
23	84	f	SDH	Left	OAK	NOAC	14	4	8	8	Left M1	Yes	No	3	CE	4	Uk
24	58	m	SDH	Uk	Trauma	VKA	8	5	17	8	Right M1	Uk	No	0	CE	5	5
25	78	m	SDH	Left	OAK	NOAC	6	4	17	9	Left ACI	Yes	No	3	CE	6	6
26	92	m	SDH	Bilateral	Unknown	No	Uk	4	25	7	Left M1	Yes	No	0	SUE	4	4
27	79	f	SDH	Right	Trauma	NOAC	10	3	8	9	Right M2	Yes	No	2b	CE	6	6
28	83	f	SDH	Uk	OAK	Yes	8	3	8	6	Left ACI	Uk	No	2b	CE	5	Uk
29	75	m	SDH	Right	Uk	No	Uk	4	24	8	Left M1	No	No	3	LAAS	4	5
30	80	f	SDH	Bilateral	Uk	No	Uk	0	42	NA	BA/VA	NA	No	0	LAAS	6	6
31	72	m	SDH	Left	Trauma	VKA	5	0	4	9	Right M1	No	No	2b	CE	2	3
32	64	m	SDH	Right	OAK	Yes	28	3	22	10	BA	NA	No	3	CE	6	6

*ICH* intracerebral hemorrhage, *SAH* subarachnoid hemorrhage, *SDH* subdural hematoma, *Uk* unknown, *NA* not applicable, *IVT* intravenous thrombolysis, *NOAC* novel oral anticoagulant, *VKA* vitamin K antagonist, *OAC* oral anticoagulation, *NIHSS* National Institute of Health Stroke Scale, *ASPECTS* Alberta stroke programme early CT score, *M1/M2* respective segment of the medial cerebral artery, *ACI* terminal internal carotid artery, *BA* basilar artery, *VA* vertebral artery, *mTICI* modified treatment in cerebral infarction scale, *mRS* modified Rankin scale, *CE* cardioembolism, *SUE* Stroke of Unknown Etiology, *LAAS* large-artery atherosclerosis

Interval indicates the time interval between onset of ICH and LVO in days (time interval between occurance of the hemorrhage and occurance of the ischemic stroke)

^a^ indicates LVO onset before ICH, and

^b^ indicates unknown sequence of same-day ICH onset and LVO

The most frequent form of intracranial hemorrhage was SAH in 40.6%, followed by ICH in 37.5% and SDH in 31.1% of cases, with 9.4% of patients having more than one form of intracranial hemorrhage.

Intracranial hemorrhage was ipsilateral (or bilateral) to LVO in 67% and contralateral in 33% of cases. Among ipsilateral intracranial hemorrhage cases, LVO affected the same territory as the hemorrhage in a single case (#3): the patient experienced hemorrhagic transformation after MCA occlusion treated with MT. Reocclusion occurred 7 days later and was successfully treated by MT again. The ICH remained stable on follow-up CT but decompressive hemicraniectomy was necessary due to malignant media infarction.

Arterial hypertension was the most frequent cardiovascular risk factor (CVRF) present in 87.5% of cases, followed by atrial fibrillation (65.6%), dyslipidemia (37.5%) and diabetes (25.0%). Oral anticoagulation was taken by 37.5% of patients, of whom 4 patients were on low molecular weight heparin at the time of AIS and 28.1% of patients received antiplatelet medication.

The most frequently reported cause of bleeding was blunt trauma in 34.4% of cases, and bleeding was assumed to result from the presence of risk factors for hemorrhage, namely anticoagulation in 25% and IVT in 18.8%. Of the patients two had parenchymal hemorrhagic transformation of cerebral ischemia, one patient experienced multiple ICH due to cerebral cavernoma and one patient suffered from aneurysmatic SAH. The cause of bleeding was unknown in three patients. Among patients with a known onset of intracranial hemorrhage, it was on the same day as LVO in 46.4% (13/28 cases) and among these cases mostly attributable to IVT or trauma (46.2% and 30.8%, respectively). In the remaining 15 cases, the hemorrhage occurred at a median of 8 days (IQR, 6.5–10 days) before AIS, and LVO was attributable to the pausation of oral anticoagulation due to hemorrhage in 10/15 patients. LVO occurred before bleeding in 34%, afterwards in 56% and with indeterminable sequence on the same day in 9% of cases.

The most frequent stroke etiology according to the TOAST classification was cardioembolism in 75% of cases. The median NIHSS was 15 (IQR, 5–21), and among patients with anterior circulation LVO, cerebral imaging revealed a median ASPECT score of 9 (IQR 8-10). The occluded vessel was located in the anterior circulation in 81.3% and in the posterior circulation in 18.8% of cases. The median number of thrombectomy maneuvers was 2 (IQR, 1‑3), and successful reperfusion, defined as a modified Thrombolysis in Cerebral Infarction scale (mTICI) score of ≥ 2b, was achieved in 81.3%. Details on preprocedural and periprocedural times as well as clinical characteristics are given in Table 1 and the supplemental material.

In-hospital mortality was 25%, and 90-day mortality, which was available in 28 patients, was 50%. Functional independence at 90-day follow-up was achieved in 21.4% (6/28) of all patients and in 40% (6/15) of patients with a baseline mRS of 0–2. None of the patients with unsuccessful reperfusion (mTICI ≤ 2a) reached functional independence and 66.7% of them died (4/6).

We performed propensity score matching to generate a control group of patients from the GSR-ET without intracranial hemorrhage matched for age, sex, NIHSS and premorbid mRS. Group comparison revealed no significant difference between patients with and without preinterventional intracranial hemorrhage regarding baseline characteristics, procedural and clinical outcomes (Table 2, supplemental table 3).

**Table 2:** Baseline characteristics, periprocedural and outcome results of patients with concomitant intracranial hemorrhage and matched controls

	Intracranial hemorrhage (*n* = 32)	No hemorrhage (*n* = 128)	*P*
Median age, years (IQR)	79.0 (8.1)	80.4 (10.5)	0.47
Female sex, % (n)	50.0 (16)	56.3 (72)	0.56
Median prestroke mRS (IQR)	1.5 (0–3.25)	2 (0–3)	0.99
Median NIHSS (IQR)	15 (8–22)	15 (11.75–19.25)	0.99
Median ASPECTS (IQR)	9 (8–10)	9 (7–10)	0.31
*Cardiovascular risk factors, % (n)*			
Hypertension	87.5 (28)	81.9 (104)	0.60
Diabetes	25.0 (8)	28.1 (36)	0.83
Dyslipidemia	37.5 (12)	38.3 (49)	1.0
Atrial fibrillation	68.8 (22)	57.0 (73)	0.31
Smoking	3.2 (1)	11.6 (14)	0.31
*Current medication, % (n)*			
Antiplatelet therapy	28.1 (9)	33.6 (43)	0.67
Oral anticoagulation	37.5 (12)	27.3 (35)	0.28
*Occluded vessel, % (n)*			
Posterior circulation	18.8 (6)	12.0 (15)	0.38
Anterior circulation	81.2 (26)	88.0 (110)	0.38
*Periprocedural results*			
IVT, % (*n*)	21.9 (7)	40.6 (52)	0.065
mTICI ≥ 2b, % (*n*)	81.2 (26)	81.9 (104)	1.0
Passages, *n* (IQR)	2 (1–3)	2 (1–3)	0.84
Median time GRO to FLR, minutes (IQR)	37.5 (26.5–69.75)	46 (27–69.5)	0.87
Periprocedural adverse events, % (*n*)	28.1 (9)	26.6 (34)	0.83
New intracranial hemorrhage within 24 h, % (*n*)	6.9 (2)	10.2 (13)	0.74
*Outcome*			
Median discharge NIHSS (IQR)	11 (5–21)	8 (2–16)	0.63
Median discharge mRS (IQR)	5 (2.75–5.25)	5 (3–6)	0.50
Median mRS at 90 days (IQR)	6 (3.5–6)	5 (3–6)	0.89
mRS 0–2 at 90 days, *%* (*n*)	21.4 (6)	19.1 (22)	0.78

*ASPECTS* Alberta Stroke Program Early CT Score,* IQR* interquartile range,* NIHSS* National Institutes of Health Stroke Scale,* IVT* intravenous thrombolysis,* mTICI* modified Thrombolysis in Cerebral Infarction scale, *mRS* modified Rankin Scale,* GRO* groin puncture,* FLR* flow restoration

## Discussion

Preinterventional intracranial hemorrhage is rare in patients with AIS and LVO, affecting 0.5% of cases in our registry. This setting presents a therapeutic dilemma. While IVT is obviously contraindicated in the presence of intracranial hemorrhage (or preceded ICH in the case of IVT-associated hemorrhage), it is at the treating physician’s discretion whether the benefit of MT outweighs the risk of hemorrhage progression or to accept the known detrimental outcome of conservatively treated LVO [6].

We have here reported the largest cohort of patients undergoing MT due to LVO with preinterventional intracranial hemorrhage. Importantly, we found that MT is technically feasible in this setting, with recanalization rates similar to those reported for LVO patients without hemorrhage [6]. Secondly, 40% of successfully treated patients remained functionally independent, whereas morbidity and mortality were high in patients with unsuccessful recanalization. Of note, the pretreatment functional status was often poor as more than half of the patients were functionally dependent before LVO, which is reflected by the high mortality of 50%. Compared to a matched control cohort of patients without intracranial hemorrhage, periprocedural and clinical results were similar. Hence, MT is technically feasible and potentially beneficial in LVO with pre-existing intracranial hemorrhage.

As mentioned above, the four main types of intracranial hemorrhage differ in etiology, management and prognosis and need separate evaluation. An EDH is caused by meningeal artery or less frequently venous bleeding into the epidural space, occurring classically after blunt head trauma. On theoretical grounds, MT seems safe in patients with AIS due to LVO and preinterventional EDH due to the differing vascular territory, but no patient with EDH was included in our cohort.

An SDH occurs in general due to bleeding from the cortical bridging veins into the arachnoid space, most commonly after blunt head trauma, but also spontaneously. As MT theoretically affects only the arterial pressure in the affected territory, MT appears safe in patients with LVO and preinterventional SDH; however, among the 10 cases reported here, most patients (80%) were already functionally dependent before, and morbidity and mortality were high.

We report one case of aneurysmatic SAH referred for aneurysm coiling, where concomitant LVO of the basilar artery was detected and treated during the same intervention but the detrimental outcome precludes any further conclusions. Non-aneurysmatic SAH conveys a better prognosis and is most commonly caused by head trauma, but may also occur in AIS, especially with LVO [25, 27–29]. Among 12 cases reported here, functional independence was achieved in 3 cases.

An ICH can occur due to a variety of reasons, including ischemic stroke with hemorrhagic transformation or trauma, further risk factors include cerebral amyloid angiopathy, anticoagulation, or IVT [30]. This heterogeneous etiology is reflected in our cohort. Previous reports focused on IVT-associated ICH, which are more frequently detected in patients treated under the drip-and-ship paradigm [14, 15]. While a detrimental outcome has been reported with a mortality of 4/6 cases, mortality was markedly lower in our cohort of IVT-associated ICH, despite a comparable distribution of age and gender [14, 15]. Among patients with ICH due to oral anticoagulation, mortality was higher.

In the setting of LVO of the supplying artery of the ICH-affected territory, MT might lead to hematoma expansion, compromising neighboring non-ischemic parenchyma or ultimately leading to brain herniation, negatively affecting the patient’s prognosis. In this scenario, established predictors of ICH expansion such as the spot sign are not evaluable, further complicating decision making [31]. We identified one patient with ICH in the LVO-affected territory: hemorrhagic transformation due to LVO was followed by reocclusion of the middle cerebral artery 7 days later, treated with successful reperfusion without hematoma enlargement on follow-up imaging. This case suggests that MT might be feasible in same territory ICH and LVO, but further data are needed to guide decision-making in this setting.

The most important limitation of our study is the lack of a control group without MT, leaving the impact of withholding or performing MT in patients with concomitant bleeding unknown. Another limitation is the nature of a retrospective analysis of the prospectively collected registry. The incidence of LVO with concomitant intracranial hemorrhage might be underestimated as only patients receiving MT were included in the registry. Furthermore, some data (3.3% of values) were missing and details about further clinical management, including the timing of oral anticoagulation resumption in patients with atrial fibrillation, are unknown.

## Conclusion

The use of MT in acute stroke patients with LVO and concomitant intracranial hemorrhage is rare but appears to be feasible and potentially beneficial. Further data are needed to guide decision-making in the setting of same territory ICH and LVO.

## Supplementary Information

Supplementary tables 1-3: further baseline and periprocedural characteristics; characteristics of patients with intracerebral, subarachnoid and subdural hemorrhage; periprocedural adverse events for patients with intracranial hemorrhage and matched controls
