# Supplementary material for: Mechanical Thrombectomy in Patients with Acute Ischemic Stroke and Concomitant Intracranial Hemorrhage
Source: Clin Neuroradiol. 2022 Jan 6;32(3):809–16. doi: 10.1007/s00062-021-01128-9 (PMC9424164; doi:10.1007/s00062-021-01128-9)
Supplement: Supplementary file 1 — Supplementary tables 1-3: further baseline and periprocedural characteristics; characteristics of patients with intracerebral, subarachnoid and subdural hemorrhage; periprocedural adverse events for patients with intracranial hemorrhage and matched controls [file 62_2021_1128_MOESM1_ESM.docx]

**Supplemental Material**

Mechanical thrombectomy in patients with acute ischemic stroke and concomitant intracranial hemorrhage

**Supplementary table 1.** Further baseline and periprocedural characteristics.

*LWMH, low weight molecular heparin bridging; APT, antiplatelets therapy; AF, atrial fibrillation; SO, symptom onset; ADM, admission; LSW, last seen well; GRO, groin puncture; FLR, flow restoration; NIHSS, National Institutes of Health Stroke scale; modified Rankin scale; uk, unknown; NA, not applicable.*

|  | ***LMWH*** | ***APT*** | ***Hyper­tension*** | ***Diabetes*** | ***Dyslipid­emia*** | ***AF*** | ***SO to ADM (min)*** | ***LSW to ADM (min)*** | ***ADM to GRO (min)*** | ***GRO to FLR (min)*** | ***number of passages*** | ***discharge NIHSS*** | ***24h NIHSS*** | ***24h mRS*** |
| --- | --- | --- | --- | --- | --- | --- | --- | --- | --- | --- | --- | --- | --- | --- |
| ***1*** | no | no | yes | no | no | yes |  | uk | 235 | 25 | 4 | NA | 28 | 5 |
| ***2*** | no | no | yes | no | yes | yes |  | uk | uk | NA | uk | NA | 15 | 5 |
| ***3*** | no | yes | yes | yes | no | yes | 978 |  | 617 | 30 | 2 | 26 | 42 | 5 |
| ***4*** | NA | no | yes | no | yes | yes | 236 |  | 29 | 28 | uk | 0 | 0 | 0 |
| ***5*** | NA | yes | yes | no | yes | yes |  | 413 | 37 | uk | 2 | 24 | 20 | 5 |
| ***6*** | NA | no | yes | no | no | yes |  | 292 | 38 | 93 | 5 | 0 | 5 | 3 |
| ***7*** | NA | yes | yes | yes | no | no | 77 |  | 123 | 40 | 1 | 6 | 7 | 3 |
| ***8*** | NA | no | yes | yes | yes | yes | 276 |  | 24 | 155 | 5 | 22 | 22 | 5 |
| ***9*** | NA | no | yes | no | no | yes |  | 79 | 151 | uk | 1 | uk | uk | 5 |
| ***10*** | NA | no | yes | no | no | no |  | 310 | 130 | 26 | 1 | 6 | 6 | 2 |
| ***11*** | no | no | yes | no | yes | yes |  | 54 | 71 | 120 | 5 | 22 | 17 | 5 |
| ***12*** | NA | no | yes | no | no | no |  | 75 | 280 | 36 | 2 | uk | uk | 5 |
| ***13*** | NA | yes | yes | yes | yes | yes | 213 |  | 24 | 63 | uk | 11 | 15 | 5 |
| ***14*** | NA | no | yes | no | no | yes |  | 358 | 17 | 45 | 3 | 5 | 2 | 2 |
| ***15*** | NA | yes | yes | no | no | no | 288 |  | 247 | uk | 1 | NA | 36 | 5 |
| ***16*** | no | no | yes | no | no | yes |  | 495 | 93 | NA | 0 | NA | 36 | 6 |
| ***17*** | NA | yes | yes | yes | yes | no | 159 |  | 171 | NA | 0 | 16 | 18 | 5 |
| ***18*** | NA | no | no | no | no | no |  | 1079 | 84 | 120 | 2 | 1 | 3 | 2 |
| ***19*** | NA | no | no | no | no | yes | 268 |  | 62 | 94 | 3 | 13 | 6 | 4 |
| ***20*** | NA | no | yes | no | no | yes |  | 130 | 90 | 37 | 2 | 21 | 28 | 5 |
| ***21*** | NA | yes | yes | no | yes | yes |  | uk | 362 | uk | 1 | uk | 14 | 5 |
| ***22*** | NA | yes | yes | no | no | no |  | 182 | 63 | 26 | 2 | 4 | 10 | 3 |
| ***23*** | yes | no | yes | yes | yes | yes |  | 250 | 45 | 38 | 1 | 3 | 7 | 5 |
| ***24*** | yes | no | no | no | no | no |  | 129 | 81 | NA | 4 | 15 | 15 | 5 |
| ***25*** | yes | no | yes | yes | no | no |  | uk | uk | 21 | 3 | NA | 24 | 5 |
| ***26*** | NA | no | yes | no | yes | yes | 87 |  | 48 | NA | 0 | 11 | uk | 5 |
| ***27*** | yes | no | yes | no | no | yes | 51 |  | 59 | 28 | uk | NA | 2 | 3 |
| ***28*** | no | no | yes | no | no | yes |  | 313 | 17 | 72 | 2 | 25 | 32 | 5 |
| ***29*** | NA | yes | yes | no | yes | no | 115 |  | 52 | 13 | 1 | 9 | 12 | 5 |
| ***30*** | NA | no | no | no | no | no |  | 244 | 156 | NA | uk | NA | 42 | 5 |
| ***31*** | no | no | yes | yes | no | yes | 0 |  | 132 | 18 | 1 | 6 | 6 | 5 |
| ***32*** | no | no | yes | no | yes | yes | 0 |  | 84 | 57 | 3 | NA | 20 | 5 |

**Supplementary table 2:** Baseline characteristics, periprocedural and outcome results for patients with acute ischemic stroke and concomitant intracranial hemorrhage.

*3 patients with both ICH and SAH are included in columns indicated by ^#^. ICH, intracerebral hemorrhage; SAH, subarachnoid hemorrhage; SDH, subdural hemorrhage;* *ASPECTS, Alberta Stroke Program Early CT Score; IQR, interquartile range; NIHSS, National Institutes of Health Stroke Scale; IVT, intravenous thrombolysis; mTICI, modified Treatment in Cerebral Infarction scale; mRS, modified Rankin Scale; GRO, groin puncture; FLR, flow restoration; min, minutes.*

|  | ICH  (n=12)^#^ | SAH  (n=13)^#^ | SDH  (n=10) |
| --- | --- | --- | --- |
| Median age, years (IQR) | 80.5 (5.7) | 79.9 (8.1) | 76.5 (9.9) |
| Female sex, % (n) | 50 (6) | 62.5 (5) | 40 (6) |
| Median prestroke mRS (IQR) | 0 (0-2.25) | 0 (0-3) | 3.5 (3-4) |
| Median NIHSS (IQR) | 15.5 (9.5-20.5) | 15 (10-16) | 17 (8-23.5) |
| Median ASPECTS (IQR) | 9 (8-19) | 9 (8-10) | 8 (8-9) |
| Cardiovascular risk factors, % (n) |  |  |  |
| Hypertension | 100 (12) | 84.6 (11) | 80 (8) |
| Diabetes | 25 (3) | 15.4 (2) | 30 (3) |
| Dyslipidemia | 41.7 (5) | 30.8 (4) | 40 (4) |
| Atrial fibrillation | 75 (9) | 53.8 (7) | 60 (6) |
| Current medication, % (n) |  |  |  |
| Antiplatelet therapy | 25 (3) | 38.5 (5) | 10 (1) |
| Oral anticoagulation | 33.3 (4) | 15.4 (2) | 70 (7) |
| Main cause or risk factor for bleeding, %(n) |  |  |  |
| Ischemia | 16.7 (2) | 0 | 0 |
| IVT | 33.3 (4) | 23.1 (3) | 0 |
| Oral anticoagulation | 25 (3) | 15.4 (2) | 40 (4) |
| Trauma | 16.7 (2) | 53.8 (7) | 30 (3) |
| Other | 8.3 (1) | 7.8 (1) | 0 |
| Median time from bleeding to ischemia, days (IQR) | 0 (0-7) | 0 (0-0.25) | 8 (7-12) |
| Occluded vessel, % (n) |  |  |  |
| Posterior circulation | 16.7 (2) | 15.4 (2) | 20 (2) |
| Anterior circulation | 83.3 (10) | 84.6 (11) | 80 (8) |
| among these: hemorrhage ipsilateral | 60 (6) | 63.6 (7) | 50 (5) |
| Periprocedural results |  |  |  |
| IVT, % (n) | 41.7 (5) | 30.8 (4) | 0 |
| mTICI≥2b, % (n) | 91.7 (11) | 84.6 (11) | 70 (7) |
| Passages, n (IQR) | 2 (1.25-4.75) | 2 (1-2.25) | 1.5 (1-3) |
| Median time GRO to FLR, min (IQR) | 36 (28-93) | 45 (36-94) | 28 (19.5-47.5) |
| Outcome |  |  |  |
| Median discharge NIHSS (IQR) | 14 (45-22.5) | 11 (5-16) | 10 (6.75-14) |
| Median discharge mRS (IQR) | 5 (2-5) | 4 (4-5) | 5 (4-6) |
| Median mRS at 90 days (IQR) | 5.5 (1.25-6) | 5.5 (3.5-6) | 5.5 (4.75-6) |
| mRS 0-2 at 90 days, % (n) | 40 (4) | 25 (3) | 0 |

**Supplemental Table 3**: Full description of periprocedural adverse events for patients with concomitant intracranial hemorrhage and matched controls.

|  | **Intracranial hemorrhage**  (n=32) | **No hemorrhage**  (n=128) | **P** |
| --- | --- | --- | --- |
| Device malfunction, % (n) | 0 (0) | 1.6 (2) | 1.0 |
| Dissection or perforation, % (n) | 3.4 (1) | 4.7 (6) | 1.0 |
| Clot migration or distant embolia, % (n) | 0 (0) | 4.7 (6) | 0.59 |
| Intracranial hemorrhage, % (n) | 3.4 (1) | 3.1 (4) | 1.0 |
| Vasospasm, % (n) | 0 (0) | 6.2 (8) | 0.35 |
| Other, % (n) | 6.3 (2) | 3.1 (4) | 0.34 |
| Other – specified, (n) | Periinterventional rethrombosis (2) | Periinterventional rethrom­bosis (1), subarachnoid hemorrhage (3) |  |
